# Immune Checkpoint Inhibitor-Associated Immune-Mediated Nephropathy: A Real-World Pharmacovigilance Study

**DOI:** 10.3390/jcm15103812

**Published:** 2026-05-15

**Authors:** Aydan Mutis Alan, Ahmet Murt, Mevlüt Tamer Dinçer, Sinan Trabulus, Özkan Alan, Mehmet Rıza Altiparmak

**Affiliations:** 1 Division of Nephrology, Department of Internal Medicine, Cerrahpasa Medical Faculty, Istanbul University-Cerrahpaşa, Istanbul 34098, Turkey; ahmet.murt@iuc.edu.tr (A.M.); tamerdincer@gmail.com (M.T.D.); sinantrabulus@gmail.com (S.T.); mraltiparmak@yahoo.com (M.R.A.); 2 Division of Oncology, Department of Internal Medicine, Cerrahpasa Medical Faculty, Istanbul University-Cerrahpaşa, Istanbul 34098, Turkey; ozkan.alan@iuc.edu.tr

**Keywords:** immune checkpoint inhibitors, immune-related nephropathy, pharmacovigilance, FDA adverse event reporting system, disproportionality analysis, drug-related side effects, adverse reactions

## Abstract

**Background/Objectives:** Immune checkpoint inhibitors (ICIs) have transformed cancer therapy, providing substantial survival benefits across a wide range of malignancies. However, ICI-associated renal toxicity encompasses a broad spectrum of clinical entities, ranging from nonspecific acute kidney injury to well-defined immune-mediated nephropathies with distinct pathophysiological mechanisms. **Methods**: We performed a large-scale pharmacovigilance study using the U.S. Food and Drug Administration Adverse Event Reporting System (FAERS) database to evaluate immune-mediated nephropathy associated with ICIs from January 2014 to March 2025. To improve specificity and minimize misclassification, the analysis was restricted to well-defined immune-mediated renal adverse events identified using MedDRA Preferred Terms, excluding nonspecific acute kidney injury. Disproportionality analysis was conducted using reporting odds ratios (RORs) with 95% confidence intervals (CIs) to assess associations between individual ICIs, treatment regimens, and nephropathy reporting. **Results**: Among 203,652 ICI-related adverse event reports (irAEs), 2361 (1.12%) involved immune-mediated nephropathy. Compared with other irAEs (non-nephropathy), immune-mediated nephropathy was more frequently reported in patients aged ≥ 65 years and in those with lung and genitourinary malignancies. Tubulointerstitial nephritis was the predominant subtype. Higher reporting signals were observed with cemiplimab and pembrolizumab, whereas durvalumab and atezolizumab demonstrated lower reporting signals. Combination regimens involving PD-1 and CTLA-4 inhibitors were associated with higher reporting frequencies compared with monotherapy. **Conclusions**: This real-world pharmacovigilance analysis identifies clinically relevant differences in immune-mediated nephropathy reporting across ICI classes and treatment strategies. PD-1 inhibitors and PD-1/CTLA-4 combination regimens were associated with higher reporting signals, suggesting potential variation in renal safety profiles. These findings should be interpreted cautiously, given the inherent limitations of spontaneous reporting systems, but they provide hypothesis-generating evidence to support future prospective studies with detailed clinical and histopathological correlation.

## 1. Introduction

Immune checkpoint inhibitors (ICIs) are humanized monoclonal antibodies that enhance T cell-mediated antitumor responses by blocking inhibitory receptors such as PD-1 (programmed cell death protein 1), PD-L1 (programmed death-ligand 1), CTLA-4 (cytotoxic T-lymphocyte-associated protein 4), and LAG-3 (lymphocyte activation gene-3) [1]. Under normal physiological conditions, these immune checkpoints prevent excessive immune activation and help maintain self-tolerance. In the context of cancer, blocking these inhibitory signals enables T cell activation and facilitates the generation of an effective antitumor immune response. The advent of ICIs has revolutionized the therapeutic landscape of oncology over the past decade. The first approved agent was ipilimumab, a CTLA-4 antagonist, followed by PD-1 inhibitors such as nivolumab, pembrolizumab, and cemiplimab, as well as PD-L1 inhibitors including atezolizumab, avelumab, and durvalumab. These agents are now widely used across a growing number of solid and hematologic malignancies, not only in metastatic disease but also in adjuvant and neoadjuvant settings [2].

Despite their promising therapeutic potential, ICIs are associated with a broad spectrum of immune-related adverse events (irAEs), which result from non-specific activation of the immune system and may lead to inflammatory toxicities affecting various organ systems [3]. The incidence of irAEs among patients receiving ICIs is estimated to range from 60% to 85%, with the skin, gastrointestinal tract, lungs, liver, and endocrine glands being the most commonly affected organ systems [4,5]. Although renal involvement is less frequent compared to other irAEs, it has garnered increasing attention due to its clinical significance. ICI-associated nephrotoxicity most commonly presents as acute kidney injury (AKI), which, in severe cases, may progress to irreversible renal impairment. Reported rates of all-grade renal toxicity are approximately 2% with ICI monotherapy and may rise to 4.9% when ICIs are administered in combination therapy [6,7].

Although clinical recognition of ICI-associated renal toxicities has increased, comparative data on the renal safety profiles of different ICIs—including PD-1, PD-L1, and CTLA-4 inhibitors remain limited [7]. Real-world pharmacovigilance databases, such as the U.S. Food and Drug Administration Adverse Event Reporting System (FAERS), offer valuable insights into the incidence, clinical manifestations, severity, and outcomes of ICI-related irAEs. These large-scale, publicly available data sources enable the identification of rare, delayed, or otherwise underreported toxicities that may not be fully captured in randomized controlled trials, thus playing a critical role in post-marketing safety surveillance and guiding clinical decision-making [8].

This study aimed to systematically evaluate immune-mediated nephropathy associated with ICIs using data from the FAERS database. Unlike prior studies that primarily focused on nonspecific AKI, we specifically targeted well-defined immune-mediated nephropathies to improve diagnostic specificity and reduce misclassification bias. We further performed a comprehensive comparative analysis across individual ICIs and treatment regimens, including monotherapy and combination strategies, to identify differential reporting signals. By focusing on well-defined immune-mediated nephropathies, this study aims to provide a more precise characterization of ICI-associated nephropathies and to inform future risk stratification and prospective studies.

## 2. Materials and Methods

### 2.1. Data Source and Extraction

We conducted a retrospective pharmacovigilance study using the publicly available FAERS database. Adverse event reports submitted between 1 January 2014 and 30 March 2025 were extracted and analyzed. Data extraction and processing were performed in accordance with standard FAERS data handling procedures. The FAERS database was queried for reports involving ICIs approved by the U.S. Food and Drug Administration (FDA) as of 30 March 2025. The ICIs included anti-PD-1 agents (nivolumab, pembrolizumab, cemiplimab, dostarlimab, toripalimab, retifanlimab, and tislelizumab), anti-PD-L1 agents (atezolizumab, avelumab, and durvalumab), anti-CTLA-4 agents (ipilimumab and tremelimumab), and the anti-LAG-3 agent relatlimab.

### 2.2. Case Identification, Data Cleaning, and Study Population

The primary objective of this study was to identify immune-mediated nephropathy associated with ICIs. Adverse events were identified using Preferred Terms (PTs) from MedDRA Version 28.0 [9]. The complete list of PTs is provided in Supplementary Table S1. A predefined and conservative case definition was applied, focusing on well-defined immune-mediated renal adverse events. To improve specificity and reduce misclassification bias, nonspecific renal-related PTs—including acute kidney injury, acute renal failure, and renal failure not otherwise specified—were excluded, as these terms may represent heterogeneous and non-immune-mediated etiologies. In the FAERS database, diagnoses are based on the reporter’s clinical assessment and are coded using MedDRA Preferred Terms referred; no independent clinical or histopathological validation is available. Therefore, cases labeled as acute kidney injury may include heterogeneous etiologies, including potential immune-mediated interstitial nephritis that cannot be fully distinguished. This conservative approach, prioritizing specificity over sensitivity, was adopted to ensure accurate identification of immune-mediated nephropathies, in line with established pharmacovigilance practices. However, this strategy may have led to underestimation of the overall burden of renal adverse events by excluding nonspecific presentations. Importantly, co-reported conditions such as sepsis, hypotension, or dehydration could not be systematically excluded. Nevertheless, our restrictive MedDRA PT-based case definition, focused on well-defined immune-mediated renal diagnoses, was designed to minimize the inclusion of cases more likely attributable to hemodynamic or infectious causes rather than immune-mediated mechanisms.

A total of 215,907 Immune-related adverse event (irAE) reports were initially retrieved from the FAERS database. Duplicate entries (n = 6100) were identified and removed using FAERS case identification numbers. When multiple reports shared the same case ID, records were cross-checked for consistency in drug names, adverse event terms, patient demographics, and reporting dates, and only the most complete and most recent report was retained. However, duplicate reports submitted by different reporters that could not be linked through case identifiers may not have been fully captured. Only reports in which an ICI was designated as the primary suspect drug were included. Reports involving concomitant use of known nephrotoxic agents were reviewed on a case-by-case basis and excluded when attribution to the ICI could not be reliably established. Five ICIs (toripalimab, dostarlimab, tislelizumab, retifanlimab, and relatlimab) were excluded due to an insufficient number of immune-mediated renal adverse event reports (<10 cases), to avoid unstable and unreliable disproportionality estimates.

**Figure 1:**
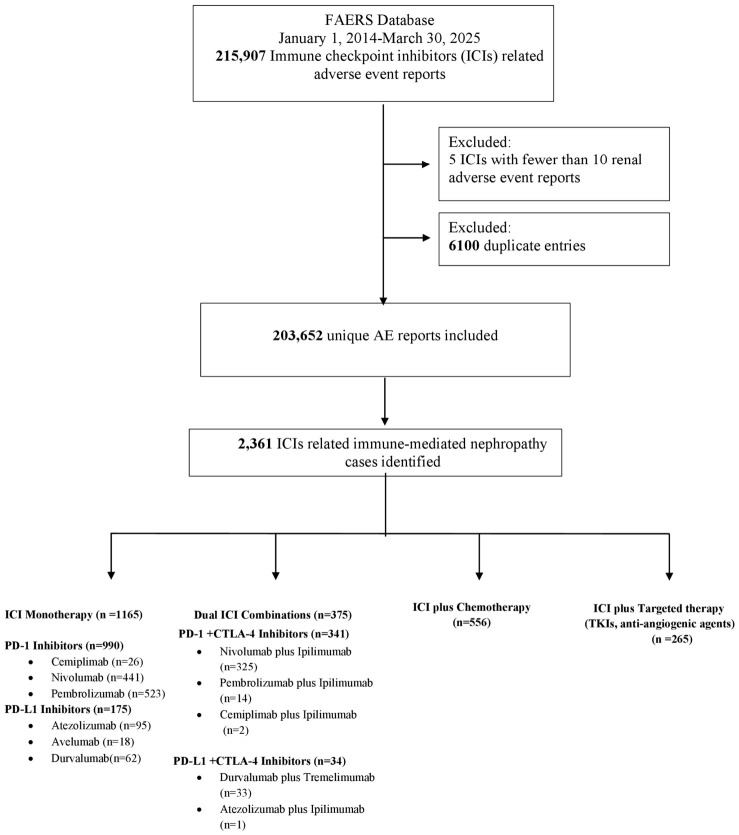
Flowchart of case selection and stratification of immune-mediated nephropathy associated with ICIs from the FAERS database. **Abbreviations:** AE: adverse event; CTLA-4: cytotoxic T-lymphocyte-associated protein; ICIs: immune checkpoint inhibitors; PD-1: programmed cell death protein 1; PD-L1: programmed death-ligand 1; TKI: tyrosine kinase inhibitor.

After these exclusions, 203,652 unique adverse event reports were included in the final analysis. Among these, 2361 cases of immune-mediated nephropathy were identified and stratified according to treatment regimen: ICI monotherapy, ICI plus chemotherapy, ICI plus targeted therapy (including agents such as tyrosine kinase inhibitors and anti-angiogenic therapies), and dual ICI combinations (PD-1 or PD-L1 inhibitors in combination with CTLA-4 inhibitors) (Figure 1).

### 2.3. Statistical and Disproportionality Analysis

Disproportionality analyses were conducted to evaluate reporting associations between ICIs and immune-mediated renal adverse events using reporting odds ratios (RORs) and corresponding 95% confidence intervals (CIs). RORs were calculated based on standard 2 × 2 contingency tables, in which reports of immune-mediated renal adverse events associated with ICIs were compared with reports of all other adverse events associated with ICIs within the FAERS database.

A signal of disproportionate reporting was considered present when the lower limit of the 95% CI exceeded 1.0. In addition, signals were considered reliable only when at least three cases (N ≥ 3) were reported for each drug–event pair, to avoid spurious associations based on small case counts. For treatment strategy comparisons, ICI monotherapy was used as the reference category. Subgroup analyses were performed to compare PD-1 inhibitors, PD-L1 inhibitors, and dual ICI combinations. Descriptive statistics were used to summarize report characteristics and the distribution of immune-mediated nephropathy. Comparisons of categorical variables were performed using the chi-square test. All analyses were descriptive and exploratory in nature and were designed to characterize reporting patterns rather than to establish causal associations. When overall comparisons were statistically significant, post hoc pairwise analyses were performed, and Bonferroni correction was applied to account for multiple comparisons. Post hoc pairwise comparisons were restricted to treatment groups with at least 30 reports to ensure statistical stability and reduce the impact of sparse data. Statistical analyses were performed using IBM SPSS Statistics for Windows, version 25.0 (IBM Corp., Armonk, NY, USA).

## 3. Results

### 3.1. Patient Characteristics and Clinical Findings in Overall Population

A total of 203,652 irAE reports were included in the final FAERS analysis, of which 2361 (1.12%) were identified as immune-mediated nephropathy. Compared with other irAE reports (non-nephropathy), immune-mediated nephropathy was more frequently reported in patients aged ≥65 years (52.9% vs. 42.1%; *p* < 0.001), with no significant difference observed between sexes (39.2% vs. 36.8% for females; *p* = 0.37). The highest reporting frequencies were observed in patients with lung or respiratory malignancies (40.7%), followed by genitourinary (15.8%) and skin/melanoma cancers (13.3%; *p* < 0.001). Compared with non-nephropathy reports, immune-mediated nephropathy was associated with higher proportions of serious outcomes (99.6% vs. 89.4%) and hospitalization (46.9% vs. 37.5%; *p* < 0.001), but a lower reported mortality rate (7.2% vs. 24.6%; *p* < 0.001). Detailed patient characteristics and clinical reporting patterns are presented in Table 1.

#### Reporting Odds Ratio (ROR) Estimates in the Overall Cohort

Compared with ICI monotherapy, combination therapy showed a numerically higher reporting frequency of immune-mediated nephropathy; however, this difference was not statistically significant (ROR: 1.06; 95% CI: 0.94–1.19). Similarly, no significant association was observed for ICI combined with targeted therapy (ROR: 0.96; 95% CI: 0.84–1.10). In contrast, ICI combined with chemotherapy was associated with a significantly lower reporting frequency of immune-mediated nephropathy (ROR: 0.88; 95% CI: 0.80–0.98). In subgroup analyses, patients aged ≥ 65 years demonstrated a significantly higher reporting frequency compared with those < 65 years (ROR: 1.26; 95% CI: 1.15–1.38), whereas no significant difference was observed between sexes (ROR: 1.04; 95% CI: 0.96–1.13). Overall, these findings suggest age-related differences in reporting patterns, without evidence of sex-based variation.

### 3.2. Comparative Analysis of Immune-Mediated Nephropathy Across ICI Regimens

**Figure 2:**
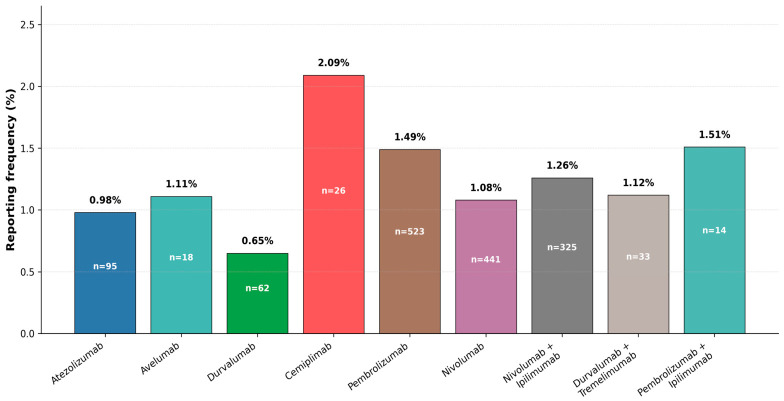
Reporting frequency of immune-mediated nephropathy across immune checkpoint inhibitor monotherapy and combination regimens in the FAERS database. Values represent the proportion of reports with immune-mediated nephropathy among all adverse event reports for each treatment. Monotherapy and combination regimens are displayed separately to highlight differences in reporting patterns across treatment strategies. Statistical comparisons were performed using the chi-square test; *p*-values reflect differences in reporting proportions and should be interpreted with caution in the context of spontaneous reporting data.

Among ICI monotherapy agents, the reporting frequency of immune-mediated nephropathy varied significantly (*p* < 0.001), ranging from 0.65% with durvalumab to 2.09% with cemiplimab. Post hoc pairwise comparisons with Bonferroni correction demonstrated that cemiplimab and pembrolizumab remained significantly associated with higher reporting frequencies compared with durvalumab, atezolizumab, and nivolumab (all adjusted *p* < 0.05). In addition, nivolumab showed a higher reporting frequency than durvalumab (adjusted *p* < 0.05). For combination regimens, reporting frequencies ranged from 1.12% to 1.51%, with no significant differences between groups (*p* = 0.63). Compared with durvalumab monotherapy, nivolumab plus ipilimumab demonstrated a significantly higher reporting frequency (*p* < 0.001), whereas no significant differences were observed between combination regimens and other ICI monotherapies. A detailed overview of immune-mediated nephropathy across treatment regimens is presented in Figure 2. Overall, these findings highlight variability in reporting patterns across individual ICIs, particularly among PD-1 inhibitors.

#### Reporting Odds Ratio Estimates by ICI Treatment Regimen

**Figure 3:**
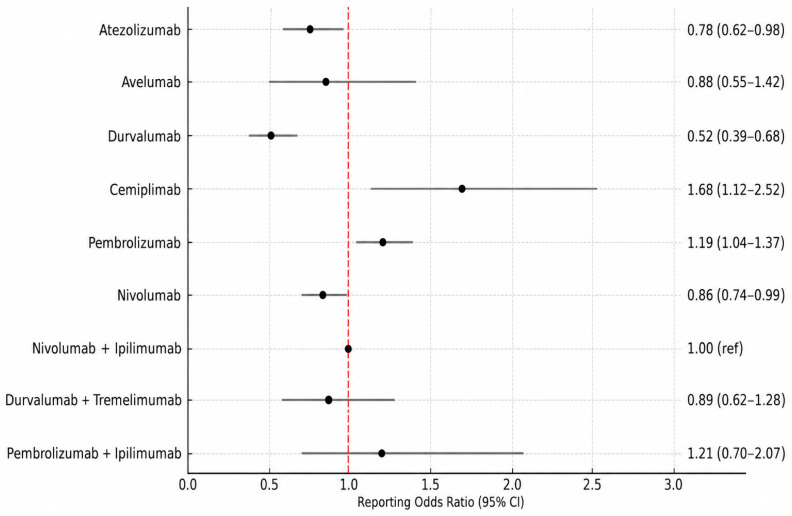
Reporting odds ratios (RORs) of immune-mediated nephropathy across individual immune checkpoint inhibitors, using nivolumab plus ipilimumab as the reference regimen in the FAERS database. Error bars represent 95% confidence intervals; the vertical dashed line indicates the reference value (ROR = 1.0).

Using nivolumab plus ipilimumab as the reference regimen, selected due to its widespread clinical use and relatively higher number of reports compared with other dual ICI combinations, cemiplimab (ROR: 1.68; 95% CI: 1.12–2.52) and pembrolizumab (ROR: 1.19; 95% CI: 1.04–1.37) were associated with significantly higher reporting frequencies of immune-mediated nephropathy. In contrast, significantly lower reporting frequencies were observed for durvalumab (ROR: 0.52; 95% CI: 0.39–0.68), atezolizumab (ROR: 0.78; 95% CI: 0.62–0.98), and nivolumab (ROR: 0.86; 95% CI: 0.74–0.99) (Figure 3).

**Figure 4:**
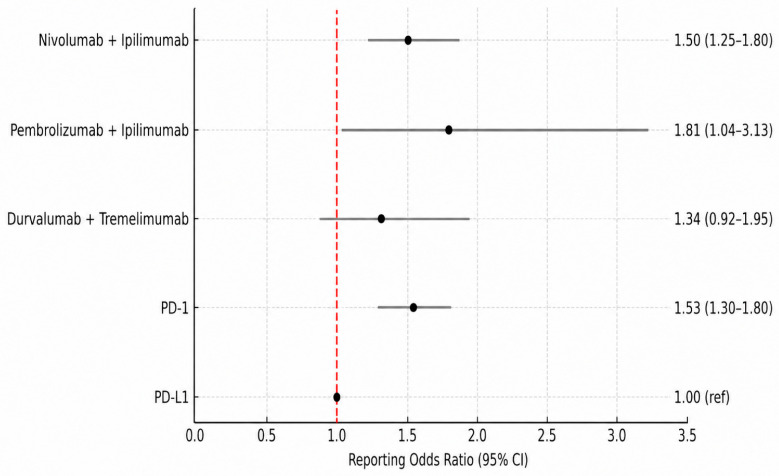
Reporting odds ratios (RORs) of immune-mediated nephropathy stratified by ICI class and treatment strategy. PD-L1 inhibitor monotherapy was used as the reference group in the FAERS database. Error bars represent 95% confidence intervals; the vertical dashed line indicates the reference value (ROR = 1.0).

In this class-based analysis, PD-L1 inhibitors were used as the reference group due to their comparatively lower reporting frequencies and their role as a clinically relevant comparator. When stratified by ICI class and treatment strategy, PD-1 inhibitors (ROR: 1.53; 95% CI: 1.30–1.80), nivolumab plus ipilimumab (ROR: 1.50; 95% CI: 1.25–1.80), and pembrolizumab plus ipilimumab (ROR: 1.81; 95% CI: 1.04–3.13) demonstrated higher reporting frequencies compared with PD-L1 inhibitors (Figure 4). Overall, these findings suggest distinct differences in reporting patterns across ICI classes and treatment strategies, with higher signals observed for PD-1–based therapies and selected combination regimens. ROR values reflect disproportionality in reporting and do not represent absolute risk or establish causal relationships.

### 3.3. Distribution of Immune-Mediated Nephropathy Subtypes Across ICI Regimens

Tubulointerstitial nephritis (TIN) was the most frequently reported subtype of immune-mediated nephropathy across all ICI regimens, accounting for 58.6% of cases overall. Distinct differences were observed in the distribution of immune-mediated nephropathy subtypes across treatment strategies. Renal vascular events were most frequently reported in the ICI plus targeted therapy group (4.2%), whereas nephritic syndrome was most common in dual ICI combinations (43.7%). Nephrotic syndrome was also most frequently observed in the ICI plus targeted therapy group (12.5%), while rapidly progressive glomerulonephritis (RPGN) was more commonly reported in ICI monotherapy (2.5%). Significant differences in the distribution of immune-mediated nephropathy subtypes were observed across treatment regimens (*p* < 0.001). However, post hoc analyses did not demonstrate a significant difference between ICI monotherapy and ICI combined with targeted therapy (*p* > 0.05). Detailed comparisons are presented in Table 2, and the overall spectrum of immune-mediated nephropathy is summarized in Table S2. These findings highlight heterogeneity in the spectrum of immune-mediated nephropathy across different ICI treatment strategies.

**Figure 5:**
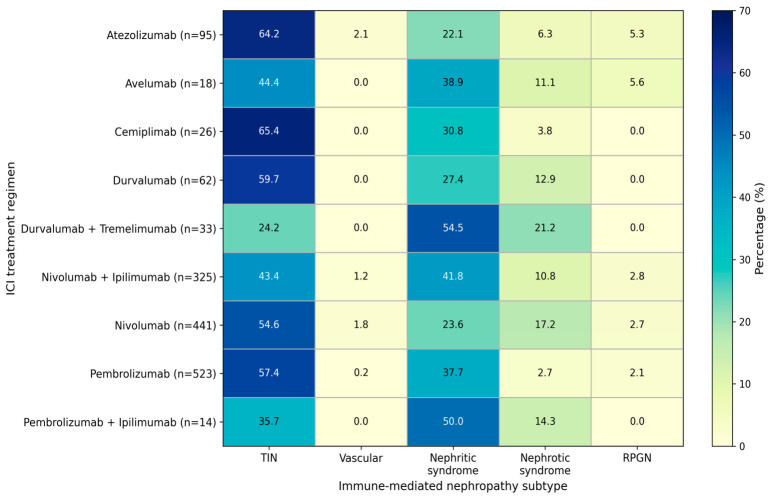
Distribution of immune-mediated nephropathy subtypes across immune checkpoint inhibitor treatment regimens in the FAERS database. Values represent the percentage distribution of nephropathy subtypes within each treatment group. Color intensity reflects the proportion (%) of each nephropathy subtype. Abbreviations: TIN, tubulointerstitial nephritis; RPGN, rapidly progressive glomerulonephritis.

Further stratified analyses of ICI monotherapy and CTLA-4–based combination regimens confirmed that TIN remained the predominant subtype of immune-mediated nephropathy. In contrast, nephritic syndrome was more frequently reported in combination regimens, particularly with durvalumab plus tremelimumab (54.5%) and pembrolizumab plus ipilimumab (50.0%). Nephrotic syndrome showed higher reporting proportions in the durvalumab plus tremelimumab (21.2%) and nivolumab monotherapy (17.2%) groups. Significant differences were observed among treatment groups (*p* < 0.05). Post hoc pairwise comparisons restricted to groups with ≥30 reports to ensure more stable estimates demonstrated that atezolizumab differed significantly from durvalumab plus tremelimumab (*p* = 0.001), nivolumab plus ipilimumab (*p* = 0.021), and pembrolizumab (*p* = 0.013) after Bonferroni correction, based on differences in the overall distribution of immune-mediated nephropathy subtypes. The distribution of immune-mediated nephropathy subtypes across treatment regimens is presented in Figure 5.

Disproportionality analyses further evaluated differences in immune-mediated nephropathy subtypes across treatment strategies, using PD-(L)1 plus CTLA-4 combinations as the reference. TIN showed significantly higher reporting frequencies in the PD-1 (ROR: 1.83; 95% CI: 1.44–2.33) and PD-L1 (ROR: 2.17; 95% CI: 1.51–3.14) monotherapy groups. In contrast, nephritic syndrome demonstrated significantly lower reporting frequencies in the PD-1 (ROR: 0.59; 95% CI: 0.47–0.76) and PD-L1 (ROR: 0.45; 95% CI: 0.31–0.67) monotherapy groups. No statistically significant disproportionality signal was observed for nephrotic syndrome. Due to the limited number of reports, RPGN and renal vascular events were excluded from the ROR analysis.

## 4. Discussion

In this study, we identified 2361 cases of immune-mediated nephropathy among 203,652 reports of irAEs, corresponding to an overall reporting proportion of 1.12%, based on a large-scale pharmacovigilance analysis using the FAERS database. According to current literature, reported rates of all-grade renal toxicity are approximately 2% with ICI monotherapy and may increase to 4.9% with combination ICI therapy [6,7]. The lower reporting proportion observed in our analysis may be attributable to the selective case definition applied, as we deliberately focused on well-defined immune-mediated renal entities, including tubulointerstitial nephritis, glomerulonephritis, and renal vascular pathologies. This approach excluded nonspecific or non-immune-mediated causes of kidney injury, thereby reducing potential confounding and enabling a more precise characterization of immune-mediated nephropathy signals within pharmacovigilance data. Unlike prior pharmacovigilance studies that primarily focused on nonspecific AKI, our analysis specifically targeted well-defined immune-mediated nephropathies, representing a key novel contribution of the present study.

irAEs have been reported to be influenced by patient age and sex. In a systematic review by Xu et al., male sex and older age were identified as potential risk factors for ICI-related nephrotoxicity. However, their findings also revealed that female patients had a higher risk of developing acute TIN compared to males. Additionally, older patients were more likely to develop TIN, whereas younger patients showed a greater tendency to present with glomerular diseases. In contrast, a meta-analysis by Liu et al. found no significant association between sex and ICI-related AKI, although advanced age remained a consistent risk factor [10,11]. Consistent with these findings, our pharmacovigilance analysis demonstrated a significantly higher reporting frequency of ICI-associated immune-mediated nephropathy in patients aged ≥ 65 years compared with those < 65 years (52.9% vs. 32.6%; *p* < 0.001). In contrast, no statistically significant difference in reporting frequency was observed between female and male patients (39.2% vs. 59.5%; *p* = 0.37), supporting the absence of a consistent sex-based association in ICI-related nephropathy, consistent with previous findings across studies.

The risk of AKI associated with ICI therapy appears to vary depending on the underlying malignancy. Although findings across studies remain heterogeneous, the most frequently implicated cancer types include genitourinary malignancies, particularly renal cell carcinoma and gynecologic cancers [12]. In a comprehensive meta-analysis of 85 randomized controlled trials, Fei Liu et al. identified renal cell carcinoma and urothelial carcinoma as independent risk factors for ICI-related AKI [13]. Similarly, an observational study demonstrated that gynecologic malignancies were independently associated with an increased risk of developing ICI-related AKI [14].

In our pharmacovigilance analysis, the most frequently reported tumor types among cases of immune-mediated nephropathy were lung and respiratory tract malignancies (40.7%), followed by genitourinary cancers (15.8%), and skin/melanoma (13.3%), whereas gynecologic malignancies accounted for only 3% of the reports. Importantly, our analysis focused on well-defined immune-mediated renal adverse entities-such as tubulointerstitial nephritis, immune-mediated nephritis, and selected glomerular diseases- while excluding non-specific AKI reports. This more restrictive case definition may partly explain discrepancies compared with studies using broader AKI definitions. In addition, the higher reporting frequency observed in lung, genitourinary, and melanoma malignancies likely reflects the widespread clinical use of ICIs in these tumor types. Conversely, the lower representation of gynecologic cancers may be related to both lower exposure and the exclusion of alternative, non-immune-mediated causes of kidney injury, such as post-obstructive nephropathy.

Although PD-1 and PD-L1 inhibitors both target the same immune checkpoint pathway, they differ significantly in their biological effects, binding properties, and clinical outcomes. PD-1 inhibitors bind directly to the PD-1 receptor, blocking its interaction with both PD-L1 and PD-L2, whereas PD-L1 inhibitors selectively block PD-L1, preserving the PD-L2–PD-1 interaction. Emerging evidence suggests that PD-L2 may play an important role in maintaining peripheral immune tolerance and may exert a stronger inhibitory effect on T-cell activation due to its higher binding affinity to PD-1 compared with PD-L1. Therefore, the preservation of PD-L2 signaling in patients receiving PD-L1 inhibitors may provide a residual immunoregulatory pathway that limits excessive immune activation. In contrast, the broader blockade achieved by PD-1 inhibitors may lead to more extensive immune activation and a potentially higher susceptibility to irAEs, including renal involvement. Moreover, PD-L1 inhibitors also interfere with the interaction between PD-L1 and the costimulatory molecule B7-1 (CD80), which can have immunostimulatory effects via CD28 and immunoinhibitory effects through CTLA-4. These molecular distinctions may contribute to differences in toxicity profiles and could partly explain the higher reporting signals observed with PD-1 inhibitors in our analysis. However, given the inherent limitations of pharmacovigilance data, these findings should be interpreted as hypothesis-generating rather than establishing a causal relationship [15,16,17].

Previous studies have reported differences in renal toxicity profiles between ICI monotherapy and combination regimens involving chemotherapy or targeted therapies. As previously mentioned, the meta-analysis by Liu et al. demonstrated that the frequency of AKI varies across ICI regimens, with nivolumab plus ipilimumab showing the highest incidence, while PD-L1 inhibitors such as avelumab and durvalumab were associated with the lowest incidence. In the same analysis, conventional therapies were reported to be safer than several ICI regimens, including pembrolizumab, atezolizumab, and high-dose ipilimumab. In addition, according to CTCAE (Common Terminology Criteria for Adverse Events) based analyses, both all-grade and grade 3–5 AKI were more frequently observed with ICI plus chemotherapy compared with ICI monotherapy [13]. Consistently, Cortazar et al. reported a lower incidence of ICI-associated AKI with anti-PD-L1 agents compared to anti-PD-1 therapies, while persistent or permanent acute kidney injury was more commonly associated with anti-CTLA-4 agents. Furthermore, combination regimens involving both anti-CTLA-4 and anti-PD-1 inhibitors were linked to a higher overall risk of AKI compared to monotherapy [6]. In our pharmacovigilance analysis, ICI combination therapy and ICI combined with targeted therapy demonstrated numerically higher reporting frequencies of immune-mediated nephropathy compared to ICI monotherapy, although these differences did not reach statistical significance. In contrast, ICI plus chemotherapy was associated with a significantly lower reporting frequency of immune-mediated nephropathy. One possible explanation may involve the immunomodulatory effects of chemotherapy; however, this hypothesis should be interpreted with caution and cannot be directly evaluated using FAERS data.

Disproportionality analyses further revealed heterogeneity in immune-mediated nephropathy reporting across individual ICI regimens. Cemiplimab and pembrolizumab were associated with significantly higher reporting odds compared with nivolumab plus ipilimumab. However, the relatively high reporting odds ratio observed for cemiplimab should be interpreted with caution, as it was based on a limited number of reports and may therefore be less stable than estimates for agents with larger reporting volumes, particularly for newer ICIs with limited post-marketing data. In contrast, durvalumab, atezolizumab, and nivolumab monotherapy showed significantly lower reporting odds. These findings are consistent with prior observations suggesting a more favorable renal safety profile for PD-L1 inhibitors [6,15,16]. Notably, combination regimens such as pembrolizumab plus ipilimumab and durvalumab plus tremelimumab did not demonstrate statistically significant differences, which may reflect either comparable safety profiles or limited statistical power due to lower reporting numbers. When analyzed by ICI class, PD-1 inhibitors and PD-1/CTLA-4 combinations regimens were associated with higher reporting frequencies of immune-mediated nephropathy compared with PD-L1 inhibitors. Taken together, these results suggest that both ICI class and treatment strategy may influence the reporting patterns of renal immune-mediated nephropathy. It is important to emphasize that ROR values reflect disproportionality in reporting rather than absolute risk. An elevated ROR indicates a higher-than-expected reporting frequency compared with the reference group. However, given the inherent limitations of pharmacovigilance data, these findings should be interpreted as hypothesis-generating rather than definitive evidence, while supporting existing literature that favors careful consideration of PD-L1 inhibitors in patients at increased risk for nephrotoxicity.

Kidney injury may involve one or more renal compartments, including the glomeruli, proximal and distal tubules, or the interstitial tissue. Histopathological data from case series and kidney biopsies consistently indicate that TIN represents the most frequently observed lesion in ICI-associated AKI. Nevertheless, a broad spectrum of additional glomerular and tubular pathologies has been described, including thrombotic microangiopathy (TMA), pauci-immune glomerulonephritis, membranous nephropathy, minimal change disease, C3 glomerulonephritis, IgA nephropathy, lupus nephritis, focal segmental glomerulosclerosis (FSGS), amyloidosis, renal tubular acidosis, and acute tubular injury. The heterogeneity in timing of onset and histopathologic patterns observed across different ICIs may reflect distinct underlying immunologic mechanisms [6,18]. Anti-PD-1 and anti-PD-L1 therapies have most commonly been associated with tubulointerstitial nephritis, which is typically characterized by diffuse interstitial infiltration with lymphocytes (predominantly CD3^+^ and CD4^+^ T cells), eosinophils, and plasma cells. These interstitial and tubular lesions may resemble those observed in autoimmune conditions such as lupus nephritis and are often accompanied by lymphocytic infiltration and interstitial edema [19,20]. In a pharmacovigilance study by Haeuser et al., using data from VigiBase (the World Health Organization’s global pharmacovigilance database), disproportionality analysis demonstrated a significant reporting signal for nephritis associated with ICI therapy. Pembrolizumab, nivolumab, and the combination of ipilimumab plus nivolumab exhibited the highest reporting odds for nephritis [21]. Similarly, Qu et al. reported that the range and intensity of renal adverse events varied across ICIs, with nivolumab, pembrolizumab, and ipilimumab showing the broadest and strongest associations, particularly with autoimmune nephritis [12]. Consistent with these findings, our FAERS-based analysis identified TIN as the most frequently reported immune-mediated renal adverse event across ICI regimens. TIN was predominantly reported with PD-1 and PD-L1 inhibitor monotherapy, whereas nephritic syndrome was more frequently observed with combination regimens involving PD-(L) 1 and CTLA-4 inhibitors, highlighting potential differences in renal immune toxicity profiles according to treatment strategy.

This study has several limitations inherent to the use of the FAERS database. First, as a spontaneous and voluntary reporting system, FAERS is subject to substantial underreporting and reporting bias, particularly for less severe or anticipated adverse events. In addition, FAERS lacks denominator data, as the total number of patients exposed to each drug is unknown; therefore, true incidence rates cannot be calculated, and comparisons across agents should be interpreted with caution. Accordingly, higher reporting frequencies observed for certain agents may partly reflect their more widespread clinical use rather than a true increase in risk. Second, the quality and completeness of reports can vary considerably, with frequent omissions of essential clinical information such as comorbidities, dosing, timing of onset, and diagnostic confirmation. Additionally, potential duplicate entries cannot always be reliably excluded, and confounding factors—such as concomitant medications and underlying diseases—are often not systematically recorded. Furthermore, the potential influence of the Weber effect should be considered, whereby newly approved drugs may be subject to increased reporting during the early post-marketing period, potentially leading to an overestimation of adverse event signals [22,23]. Moreover, FAERS does not provide laboratory measurements (e.g., serum creatinine changes, urinalysis findings, or proteinuria levels) or detailed histopathological data. As a result, renal adverse events in this study were identified at the reporting level, precluding granular severity grading (e.g., CTCAE classification) or pathological verification, which may limit the assessment of disease severity and diagnostic certainty and thereby affect the clinical interpretation of the findings. The deliberate exclusion of non-specific renal adverse events, such as AKI, was intended to improve specificity and reduce misclassification bias. However, this approach may have reduced sensitivity and limited the external validity of the findings, as some cases of immune-mediated nephropathy presenting with nonspecific features may have been excluded. Therefore, the results should be interpreted in light of this trade-off between specificity and sensitivity. Finally, causal relationships cannot be established using FAERS data, as it represents a passive pharmacovigilance system; thus, the findings should be interpreted as hypothesis-generating signals rather than definitive evidence. Despite these limitations, a key strength of our study is its focus on well-defined, immune-mediated renal adverse events rather than non-specific AKI. By including only clinically and mechanistically distinct entities—such as tubulointerstitial nephritis, immune-mediated nephritis, and selected glomerular pathologies—we aimed to provide a more precise characterization of ICI-associated nephrotoxicity. This targeted approach enhances the interpretability and relevance of our findings by minimizing confounding from unrelated causes of AKI, such as prerenal factors, post-obstructive nephropathy, or ischemic injury.

## 5. Conclusions

This large-scale pharmacovigilance and literature-based analysis highlights the broad and clinically relevant spectrum of immune-mediated nephropathy associated with ICIs. While TIN remains the most commonly reported subtype across ICI regimens, glomerular and vascular pathologies also contribute to the heterogeneity of renal involvement. The observed differences in reporting patterns among ICI classes and treatment strategies, including the higher reporting frequency associated with PD-1/CTLA-4 combination therapies, should be interpreted in the context of pharmacovigilance data and may not directly reflect absolute incidence rates in routine clinical practice. Nevertheless, these findings provide important hypothesis-generating evidence to inform risk stratification and support the design of future prospective studies incorporating systematic histopathologic evaluation. Such studies will be essential to guide therapeutic decision-making and ensure safer ICI rechallenge protocols.

## Figures and Tables

**Table 1:** Baseline characteristics and clinical outcomes of immune-mediated nephropathy versus non-nephropathy reports.

Variables		Reports n (%)		
		Other ICI-Related Adverse Event (n:201,291)	Immune-Mediated Nephropathy (n:2361)	*p* Value
Age (year)	<65	66,035 (32.8)	772 (32.6)	<0.001
	≥65	84,825 (42.1)	1249 (52.9)	
	Not specified	50,431 (25.1)	340 (14.5)	NA
Sex	Female	74,123 (36.8)	926 (39.2)	0.37
	Male	108,233 (53.8)	1406 (59.5)	
	Not specified	18,935 (9.4)	29 (1.3)	NA
Primary Tumor Site	Lung/Respiratory System	64,572 (32.1)	961 (40.7)	<0.001
	Head and Neck	3480 (1.7)	45 (1.9)	
	Gastrointestinal	13,003 (6.5)	135 (5.7)	
	Hepatobiliary	10,753 (5.3)	108 (4.6)	
	Genitourinary	25,332 (12.6)	373 (15.7)	
	Gynecological	9163 (4.6)	70 (3.0)	
	Skin/Melanoma	23,697 (11.8)	314 (13.3)	
	Breast	5973 (3.0)	107 (4.5)	
	Hematologic	3338 (1.7)	29 (1.2)	
	Other/Not specified	41,980 (20.9)	219 (9.4)	
ICIs type	ICI monotherapy	96,755 (48.1)	1165 (49.5)	0.036
	Dual ICI combination	29,365 (14.6)	375 (15.8)	
	ICI plus ChT	52,133 (25.9)	556 (23.5)	
	ICI plus targeted therapy	23,038 (11.4)	265 (11.2)	
Reporting Period	2023–2025	72,940 (36.2)	1035 (43.8)	<0.001
	2020–2022	71,870 (35.7)	860 (36.4)	
	<2020	56,481 (28.1)	466 (19.8)	
Reporter Type	Healthcare professional	169,801 (84.4)	2301 (97.5)	<0.001
	Consumer	30,813 (15.3)	60 (2.5)	
	Not specified	677 (0.3)	0	NA
Region of Report	Africa	304 (0.2)	0	<0.001
	Asia	54,504 (27.1)	779 (33.0)	
	Europe	52,482 (26.1)	799 (33.8)	
	North America	67,050 (33.3)	625 (26.5)	
	South America	2700 (1.3)	8 (0.3)	
	Oceania	3390 (1.7)	39 (1.7)	
	Not Specified	20,861 (10.4)	111 (4.7)	NA
Outcomes	Non-serious outcomes	21,315 (10.6)	8 (0.4)	<0.001
	Serious outcomes	179,976 (89.4)	2353 (99.6)	
	Death	49,450 (24.6)	169 (7.1)	<0.001
	Hospitalization	75,434 (37.5)	1107 (46.8)	<0.001

Footnote: Values are presented as n (%). Percentages are calculated within each column based on the total number of reports. Non-nephropathy irAE reports were defined as all other irAE reports excluding immune-mediated nephropathy. “Not specified” categories are included in percentage calculations but excluded from statistical comparisons. *p*-values were calculated using the chi-square (χ^2^) test and are presented for descriptive comparison of reporting proportions only; they should be interpreted with caution, given the inherent limitations of spontaneous reporting systems and do not imply causal associations. Minor deviations from 100% may occur due to rounding. Abbreviations: ChT, chemotherapy; ICI, immune checkpoint inhibitor; irAE, ICI-related adverse event; NA, not applicable.

**Table 2:** Distribution of immune-mediated renal adverse event subtypes across ICI treatment regimens.

Treatment Type	Renal Adverse Events Reports n = 2361 (%)					
	TIN	Vascular	Nephritic Syndrome	Nephrotic Syndrome	RPGN	Total
ICI monotherapy	664 (57.0)	11 (0.9)	354 (30.4)	107 (9.2)	29 (2.5)	1165 (100)
Dual ICI combinations	154 (41.1)	4 (1.1)	164 (43.7)	44 (11.7)	9 (2.4)	375 (100)
ICI plus Targeted Therapy	144 (54.3)	11 (4.2)	76 (28.7)	33 (12.5)	1 (0.4)	265 (100)
ICI plus Chemotherapy	421 (75.7)	6 (1.1)	105 (18.9)	12 (2.2)	12 (2.2)	556 (100)

Footnote: Values are presented as n (%), calculated within each treatment group. Nephritic syndrome represents a grouped category of immune-mediated renal disorders based on MedDRA Preferred Terms; detailed definitions are provided in Supplementary Table S1. Percentages may not sum to 100% due to rounding. Dual ICI combinations, PD-1/L-1 plus CTLA-4 inhibitors. Abbreviations: CTLA-4, cytotoxic T-lymphocyte-associated protein 4; ICI, immune checkpoint inhibitor; TIN, tubulointerstitial nephritis; RPGN, rapidly progressive glomerulonephritis.

## Data Availability

The datasets generated and/or analyzed during the current study are available from the corresponding author.

## Supplementary Materials

The following supporting information can be downloaded at: https://www.mdpi.com/article/10.3390/jcm15103812/s1, Table S1: Preferred Terms (PTs) related to immune-mediated renal adverse events used in FAERS data extraction, categorized by MedDRA System Organ Class (SOC) and estimated High-Level Terms (HLTs); Table S2: Distribution of Renal Immune-related Adverse Events.

## Abbreviations

The following abbreviations are used in this manuscript:

AKI	Acute kidney injury
CI	Confidence Interval
CTLA-4	Cytotoxic T-Lymphocyte-Associated Antigen 4
FAERS	U.S. Food and Drug Administration Adverse Event Reporting System
FDA	U.S. Food and Drug Administration
ICI	Immune Checkpoint Inhibitor
ICIs	Immune Checkpoint Inhibitors
irAE	Immune-Related Adverse Event
irAEs	Immune-Related Adverse Events
LAG-3	Lymphocyte activation gene-3
MedDRA	Medical Dictionary for Regulatory Activities
PD-1	Programmed Death-1
PD-L1	Programmed Death-Ligand 1
PT	Preferred Term
RPGN	Rapidly progressive glomerulonephritis
ROR	Reporting Odds Ratio
WHO	World Health Organization
TIN	Tubulointerstitial nephritis
