# Supplementary material for: Immune Checkpoint Inhibitor-Associated Immune-Mediated Nephropathy: A Real-World Pharmacovigilance Study
Source: J Clin Med. 2026 May 15;15(10):3812. doi: 10.3390/jcm15103812 (PMC13207501; doi:10.3390/jcm15103812)
Supplement: Supplementary file 1 [file jcm-15-03812-s001.zip › table s1.pdf]

Table S1: Preferred Terms (PTs) related to immune-mediated renal adverse events used in FAERS data extraction, categorized by MedDRA System Organ Class (SOC) and estimated High-Level Terms (HLTs).

SOC	HLT (estimated)	Preferred Term (PT)
Renal and urinary disorders	Immune-mediated renal disorders	Immune-Mediated Nephritis
Renal and urinary disorders	Glomerulonephritides and nephrotic syndromes	Nephrotic Syndrome
Renal and urinary disorders	Tubulointerstitial disorders	Tubulointerstitial Nephritis
Renal and urinary disorders	Immune-mediated renal disorders	Immune-Mediated Renal Disorder
Renal and urinary disorders	Immune-mediated renal disorders	Autoimmune Nephritis
Renal and urinary disorders	Glomerulonephritides and nephrotic syndromes	Nephritic Syndrome
Renal and urinary disorders	Glomerulonephritides and nephrotic syndromes	Glomerulonephritis
Renal and urinary disorders	Other renal disorders	Glomerulonephropathy
Renal and urinary disorders	Other renal disorders	Lupus Nephritis
Renal and urinary disorders	Glomerulonephritides and nephrotic syndromes	Iga Nephropathy
Renal and urinary disorders	Glomerulonephritides and nephrotic syndromes	Igm Nephropathy
Renal and urinary disorders	Other renal disorders	Focal Segmental Glomerulosclerosis
Renal and urinary disorders	Glomerulonephritides and nephrotic syndromes	Mesangioproliferative Glomerulonephritis
Renal and urinary disorders	Glomerulonephritides and nephrotic syndromes	Glomerulonephritis Minimal Lesion
Renal and urinary disorders	Glomerulonephritides and nephrotic syndromes	Glomerulonephritis Membranous
Renal and urinary disorders	Glomerulonephritides and nephrotic syndromes	Glomerulonephritis Membranoproliferative
Renal and urinary disorders	Glomerulonephritides and nephrotic syndromes	Glomerulonephritis Proliferative
Renal and urinary disorders	Glomerulonephritides and nephrotic syndromes	Glomerulonephritis Rapidly Progressive
Renal and urinary disorders	Glomerulonephritides and nephrotic syndromes	Anti-Glomerular Basement Membrane Disease
Renal and urinary disorders	Glomerulonephritides and nephrotic syndromes	Glomerular Vascular Disorder
