# Supplementary material for: Immune Checkpoint Inhibitor-Associated Immune-Mediated Nephropathy: A Real-World Pharmacovigilance Study
Source: J Clin Med. 2026 May 15;15(10):3812. doi: 10.3390/jcm15103812 (PMC13207501; doi:10.3390/jcm15103812)
Supplement: Supplementary file 1 [file jcm-15-03812-s001.zip › TABLE s2.pdf]

Table S2) Distribution of Renal Adverse Events (n = 2361)

**Adverse Event**	**Frequency**	**Percent**
Tubulointerstitial Nephritis	1383	58.6
Immune-Mediated Nephritis	564	23.9
Nephrotic syndrome	125	5.3
IgA Nephropathy	56	2.4
Membranous nephropathy (MN)	54	2.3
Rapidly Progressive Glomerulonephritis	51	2.2
Glomerulonephritis	44	1.9
Renal Thrombotic Microangiopathy	25	1.1
Membranoproliferative Glomerulonephritis (MPGN)	14	0.6
Focal Segmental Glomerulosclerosis (FSGS)	17	0.7
Goodpasture Syndrome	10	0.4
Lupus Nephritis	8	0.3
Henoch–Schönlein Purpura	7	0.3
Fibrillary Glomerulonephritis (FGN)	2	0.1
C3 Glomerulonephritis (C3GN)	1	0.0
Total	2361	100.0
